# Grading chondroid tumors through MRI radiomics: enchondroma, low-grade chondrosarcoma and higher-grade chondrosarcoma

**DOI:** 10.1186/s12885-025-14330-6

**Published:** 2025-05-22

**Authors:** Hyerim Park, Jooyeon Lee, Seungeun Lee, Joon-Yong Jung

**Affiliations:** 1 Department of Radiology, College of Medicine, Soonchunhyang University Cheonan Hospital, Soonchunhyang University of Korea, Cheonan, Republic of Korea; 2 Department of Biostatistics and Data Science, UTHealth Houston School of Public Health, Houston, TX USA; 3 Department of Radiology, Seoul St. Mary’s Hospital, College of Medicine, The Catholic University of Korea, 222 Banpo-daero, Seocho-gu, Seoul, 06591 Republic of Korea

**Keywords:** Radiomics, Chondrosarcoma, Enchondroma, Magnetic resonance imaging, Least absolute shrinkage and selection operator.

## Abstract

**Background:**

To develop a multiclass radiomics model for differentiating chondroid bone tumors using preoperative MRI.

**Methods:**

This retrospective study included 120 patients (92 enchondromas, 16 low-grade chondrosarcomas, and 12 intermediate-to-high-grade chondrosarcomas) who underwent contrast-enhanced MRI between 2009 and 2019. Tumor segmentation was manually performed by a musculoskeletal radiologist and validated by a senior radiologist. We used least absolute shrinkage and selection operator (LASSO) and random forest (RF) for feature selection and classification, with and without synthetic minority oversampling technique (SMOTE). Model performance was evaluated using five-fold cross-validation with average precision, accuracy, area under the curve (AUC), and weighted kappa statistics.

**Results:**

The LASSO + RF model based on all sequences achieved the highest accuracy (0.826 ± 0.065) and AUC (0.967 ± 0.027). The highest mAP (0.750 ± 0.095) was observed in the SMOTE-enhanced T2WI-based model, highlighting the potential impact of class imbalance. Quadratic weighted kappa values ranged from 0.648 to 0.731 across models, indicating substantial agreement with pathological results.

**Conclusions:**

Preoperative MRI-based radiomics provides a robust method for the classification of chondroid bone tumors, potentially enhancing clinical decision-making.

## Background

Cartilaginous bone tumor is composed of chondroid matrix and tumor cells with chondrocyte differentiation [1]. The majority of cartilaginous bone tumors are enchondromas, the second most common benign bone tumors [2]. Whereas, chondrosarcoma is the third most common primary malignant tumor of the bone and is divided histologically into grade 1, grade 2, and grade 3 chondrosarcoma (CSA) [3]. The prediction of grades in cartilaginous bone tumor is of the utmost importance because therapeutic management and prognosis differ according to histologic grade [4]. Enchondroma is typically managed to a watch-and-wait approach. Grade 1 CSA generally has a good prognosis with curettage and bone graft, while grade 2 and 3 CSA have a poor prognosis even with wide resection [4, 5]. However, differentiation between grade 1 CSA and enchondroma is often difficult due to similarities in imaging and pathological features [3, 5].

In practice, MRI plays an important role in local staging of chondroid bone tumors, while CT provides complementary information such as matrix mineralization and cortical changes [6, 7]. Various radiologic features such as endosteal scalloping greater than two-third of cortical thickness, cortical destruction, soft tissue masses, and periosteal reactions are known to suggest CSA over enchondroma [8]. However, low-grade CSA often cannot be differentiated from enchondroma based only on imaging features [5]. In addition, grade 2 or 3 CSA often mimics low-grade CSA without aggressive imaging features [9, 10]. Preoperative biopsy is considered to confirm the histologic grades. However, regional biopsy sample could lead to misinterpretation of CSA grade due to the tumor heterogeneity and interobserver discrepancy for overlapping histological features [11–13].

Radiomics is a methodology to extract features including texture from images and build a machine-learning model using the features for the task at hands [14–16]. Initially, radiomics has applied for oncology field, but today it expands its horizon to inflammatory or degenerative diseases [14]. To date, several studies have applied texture analysis or radiomics on CT or MRI of cartilaginous neoplasm [12, 17–19]. Addition of radiomics features to the conventional MRI findings improved the differentiation between enchondroma and CSA [18]. Machine-learning model using radiomics features from CT or MRI showed fair diagnostic performance to differentiate low- and higher- grade CSA [17, 19, 20]. However, unlike previous studies that primarily focused on binary classification between enchondroma and low-grade CSA, or low-grade and higher-grade CSA, our study is intended to address crucial gap by developing a multiclass radiomics model designed to differentiate across the spectrum of those tumors, thereby aligning more closely with the complexity of clinical decision-making in practice.

Therefore, the purpose of our study is to develop the multiclass radiomics model to differentiate the spectrum of enchondroma, low-grade CSA and intermediate-to-high grade CSA.

## Methods

### Ethics

All procedures performed in this study were conducted in accordance with relevant guidelines and regulations, including the principles outlined in the Declaration of Helsinki. The institutional review board of Seoul St.Mary’s Hospital, The Catholic University of Korea approved this retrospective study and waived the need for informed consent.

### Study design and inclusion/exclusion criteria

We collected the data from January 2009 to August 2019 for research purpose. Patient information was retrieved through electronic medical records from the department of orthopedic surgery and pathology. The authors collected the data and anonymized the patient’s information. 158 consecutive patients who received MRI and were clinically or pathologically confirmed enchondroma or CSA were retrospectively included in our study.

The criteria for clinical confirmation of enchondroma were as follows: (1) An intraosseous mass confined to the medullary cavity, (2) Absence of cortical scalloping by the mass in long bones, (3) Presence of internal matrix calcifications with a chondroid pattern, (4) Stability in size, marginal conspicuity, and internal matrix over 2 years on serial plain radiographs. Chondroid tumors meeting all these criteria were presumed to be benign enchondromas without the need for surgical biopsy. Masses that did not fulfill the clinical criteria or displayed aggressive features such as cortical disruption, periosteal reaction, extraosseous soft tissue extension, or internal heterogeneity on initial imaging evaluation were pathologically confirmed via intralesional curettage or resection.

The inclusion criteria were (1) enchondroma or CSA that was pathologically confirmed by incisional biopsy or intralesional curettage, resection, (2) enchondroma that was clinically confirmed based on the aforementioned criteria, (3) MRI scan performed within 3 months in pathologically confirmed cases.

**Fig. 1:**
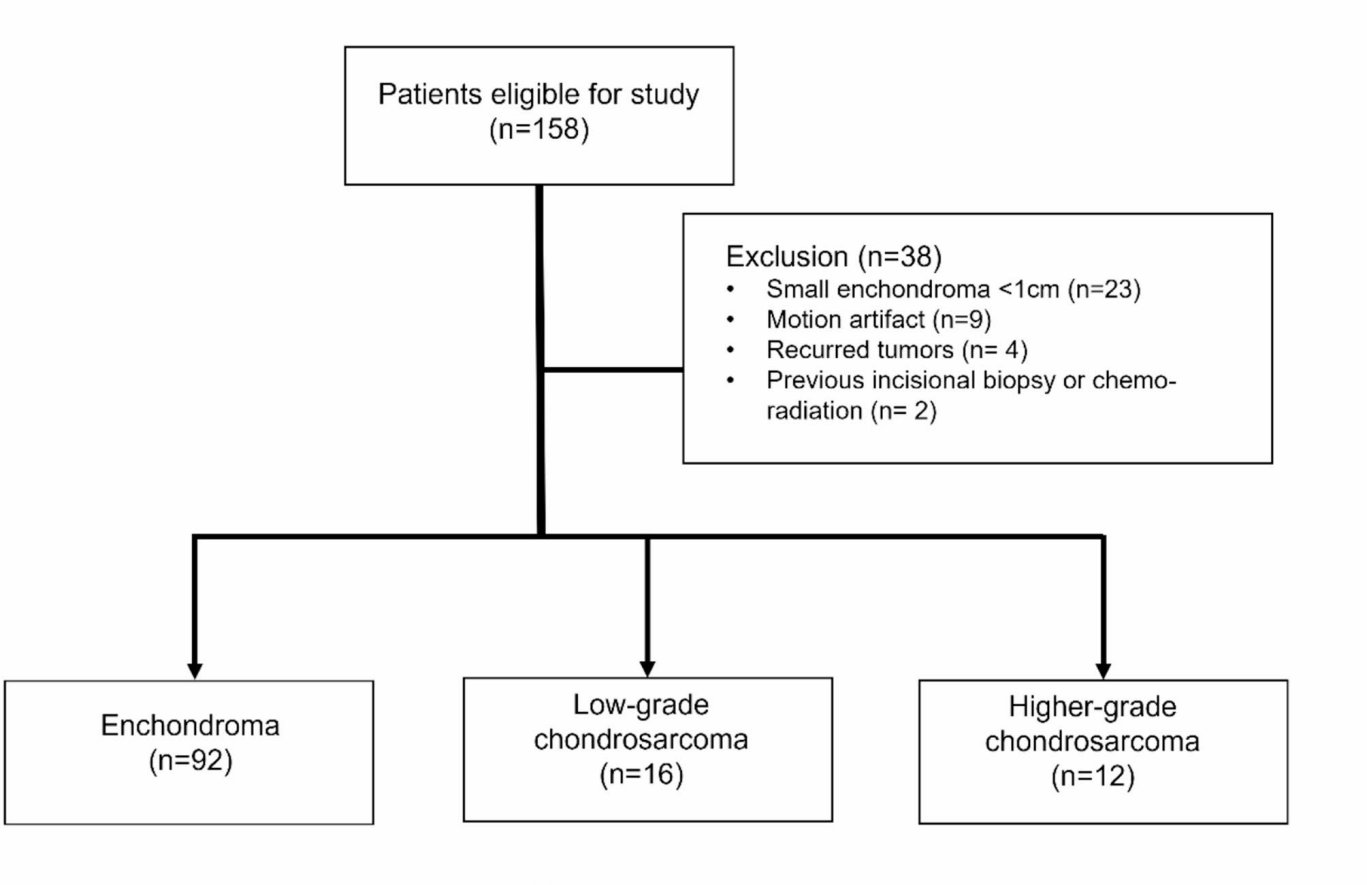
Patient Selection Flowchart for Chondroid Bone Tumor Classification

Patients with recurrent tumors, previous incisional biopsy or chemo-radiation before MRI, images with insufficient quality—such as those lacking essential sequences or affected by motion artifacts, and small enchondromas less than 1 cm were excluded. A flowchart of the patient selection process is shown in Fig. 1.

### Study cohorts

A total of 120 patients were retrospectively included in the study. Among them, 28 patients had pathologically confirmed chondroid tumors, and 92 patients had clinically diagnosed enchondromas based on the predefined clinical criteria. The mean follow-up period for these patients was 7.2 years.

Among the 120 patients included in the study, the number of patients with enchondroma, CSA grade 1, and grade 2 + 3 were 92, 16, and 12 respectively. Patient demographics, lesion locations, and grading are summarized in Table 1.

**Table 1:** Patient demographic and location of tumors

		Enchondroma (*n* = 92)	Low-grade CSA (*n* = 16)	Higher-grade CSA (*n* = 12)
Age		40 (13.8)	48(14.6)	50(15.4)
Gender (M: F)		33:59	6:10	4:8
Location	Femur	2	2	2
	Tibia	1	1	1
	Foot	14	0	0
	Pelvis	0	2	4
	Humerus	8	10	1
	Scapula	0	1	2
	Hand	67	0	0
	Rib	0	0	2

**Table 2:** MR parameters

Parameters	Axial T1-WI	Fat-suppressed axial T2-WI	Fat suppressed sagittal or coronal T2-WI	Contrast enhanced fat suppressed, axial T1-WI
**Field of view**	100–280 mm	100–280 mm	200–350 mm	100–280 mm
**Matrix size**	384 × 230 – 512 × 256	256 × 128 – 512 × 256	256 × 128 – 512 × 256	384 × 230 – 512 × 256
**TR (msec)/TE (msec)**	650–750/ 11–15	3400–4800/ 68–81	3400–4800/ 72–81	700–820/ 15–18
**Number of Slices**	25–40	25–40	25–35	25–40
**Slice thickness**	3–8 mm	3–8 mm	3–5 mm	3–8 mm
**Intersection gap**	0–0.8 mm	0–0.8 mm	0–0.8 mm	0–0.8 mm
**Turbo factor **	3	13–17	13–18	3–5
**Number of excitation**	1	1	1	1
**Fat suppression**	-	Chemical Shift Selective or Dixon-technique	Chemical Shift Selective	Chemical Shift Selective

### MRI data acquisition

MRI examinations were performed using one of two 3.0 T imagers (Verio and Magnetom Vida; Siemens Medical Solutions, Erlangen, Germany) with dedicated surface coils tailored to the tumor location. The standard MRI protocols included longitudinal fat-suppressed T2-weighted turbo spin-echo (TSE) sequence, axial T1-weighted TSE sequence, axial T2-weighted TSE sequences with and without fat suppression, and longitudinal and axial fat-suppressed contrast-enhanced T1-weighted TSE sequences. The MRI parameters for each sequence are detailed in Table 2.

### Segmentation

**Fig. 2:**
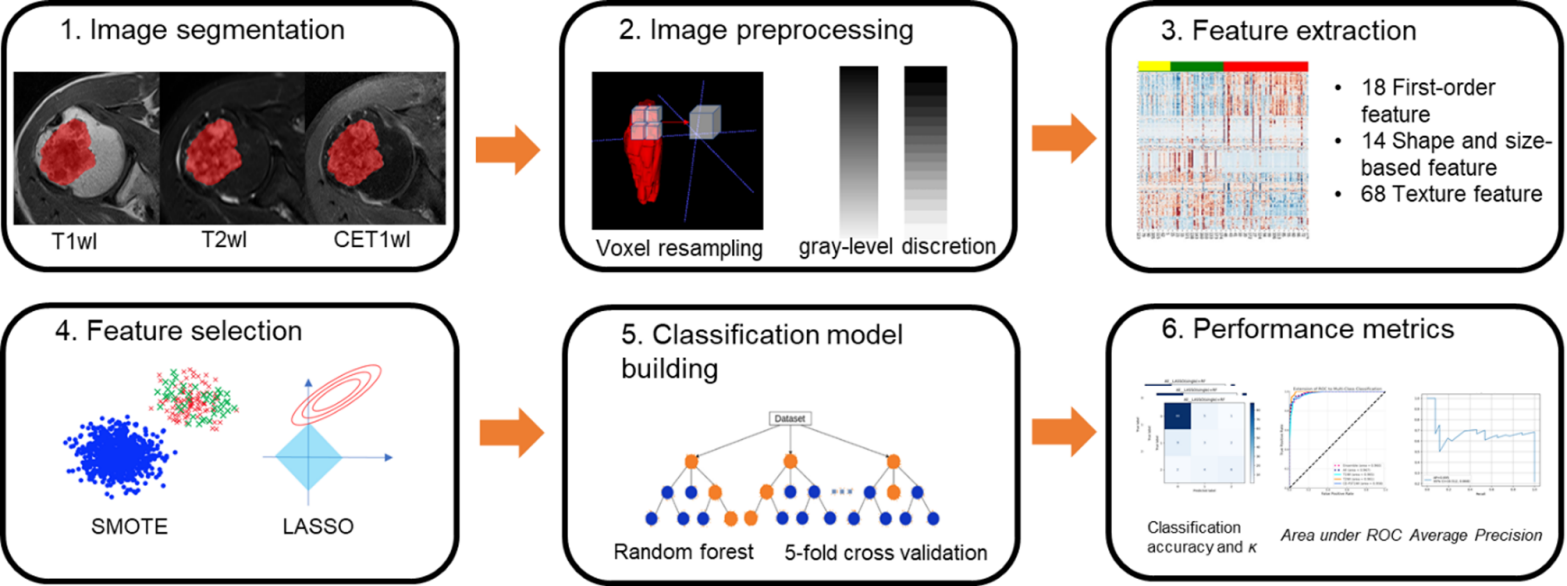
Radiomics Pipeline for Chondroid Bone Tumor Classification Using MRI Features Preoperative MR images were manually segmented, and radiomic features were extracted after standardization, including first-order, shape, and texture features. Key features were selected using the least absolute shrinkage and selection operator (LASSO) and random forest methods. Multiple classification models were developed using combinations of selected features, and five-fold cross-validation was performed to evaluate model accuracy and robustness in differentiating between enchondroma, low-grade, and higher-grade chondrosarcomas. Statistical analyses, including accuracy, weighted kappa, area under receiver-operating characteristic (ROC) curve, and average precision, were conducted to assess model performance

Lesion segmentation was performed using the open-source software ITK-SNAP (version 3.9.0, http://www.itksnap.org/) [21] (Fig. 2). The volume of interest (VOI) was manually delineated along the entire mass. A musculoskeletal radiologist with 2 years of experience (H.P) performed the initial segmentation by drawing a free-hand region of interest (ROI) along the tumor borders on T2-weighted imaging (T2WI), T1-weighted imaging (T1WI), and contrast-enhanced T1-weighted imaging (CE-T1WI) sequences, excluding any peritumoral abnormalities. The segmentations were subsequently validated by a second musculoskeletal radiologist with 16 years of experience (J.Y.J). Both radiologists were blinded to tumor entity or grade during segmentations.

### Image preprocessing and extraction of features

To minimize the intensity variations, image normalization was performed before feature extraction. During the feature extractions, image resampling and gray-level discretion were conducted. Image pre-processing and feature extraction were performed using PyRadiomics (Ver 3.0) [22]. Image preprocessing consisted in resampling to a 1 × 1 × 1 isotropic voxel (mm^3^), intensity normalization and discretization with a fixed bin width of 25. A detailed description of the implementation of these steps and radiomic features extracted by the software is available in the official documentation (https://pyradiomics.readthedocs.io/en/latest/features. html). Within each VOI, (a) 18 first-order features, (b) 14 shape and size-based features, and (c) 68 texture features were extracted. We only used original features acquired from each sequence.

### Feature selection methods and classification model Building

We used least absolute shrinkage and selection operator (LASSO) and random forest (RF) for feature selection and the development of our classification model. RF is a well-established classifier in radiomics [23]. For the 3-class classification task of RF, the predicted class was determined based on the highest predicted probability among the three classes. While RF includes its own feature selection process, LASSO is widely used due to its ability to minimize overfitting [24, 25]. We applied two different schemes for feature selection using LASSO. First, features were selected once from the entire set by LASSO, and the selected features were consistently used to train and validate each model. Second, features were repeatedly selected by LASSO for each training set during 5-fold cross-validation.

To address the issue of class imbalance, we applied the synthetic minority oversampling technique (SMOTE). This method generates synthetic examples to balance the dataset, which is known to improve the accuracy of classifiers for minority classes [26].

Ultimately, we developed 4 different models: LASSO (single) + RF, SMOTE + LASSO (single) + RF, LASSO (repeated) + RF, SMOTE + LASSO (repeated) + RF using different combination of aforementioned algorithms.

For feature input, we used various input features from T1WI, T2WI, CE-T1WI, and combination of all sequences. Additionally, we constructed an ensemble model using a weighted voting method. This ensemble combined the classification results of three RF models, each trained on features from T1, T2, and CE sequences, respectively. The highest voted group was selected based on the weights assigned to each classifier [27]. The overall steps of our radiomics pipeline are illustrated in Fig. 2.

### Statistical analysis

We assessed model performance using averaged accuracy, average precision (AP) and area under the curve (AUC) from five-fold cross-validation. The classification accuracy was used to evaluate the model’s ability to differentiate among the three grades—enchondroma, low-grade CSA, and higher-grade CSA. Since AP and AUC are not directly applicable as metrics for three-class classification, it was specifically employed for binary classification between enchondroma and CSA.

To evaluate the agreement between pathological results and the machine learning models, we applied the quadratic weighted kappa coefficient to measure the consistency among the models.

APs were calculated using Python (scikit-learn library, version 1.3.0). All other statistical analyses were performed using R version 4.0.0 (http://www.r-project.org/).

## Results

All classification models demonstrated overall mean accuracies over 0.8 and mean AP around 0.7. Among the different models, the LASSO (single) + RF model tends to demonstrated the highest accuracy, AUC and mean AP.

In the model combining LASSO (single), SMOTE, and RF, T2WI-derived features provided the highest accuracy, while the combination of all sequences resulted in the highest AUC. Mean AP was the highest for the T2WI feature set using the LASSO (single) + SMOTE + RF model. All classification models showed substantial agreements with pathologic grading, with weighted kappa values above 0.6 (Tables 3 and 4).

**Table 3:** Performance of model with single feature selection

Model	Features	Accuracy (mean ± SD)	Kappa coefficient (mean ± SD)	AUC (mean ± SD)	mAP (mean ± SD)
LASSO (single) + RF	T1WI	0.823 ± 0.060	0.648 ± 0.145	0.956 ± 0.035	0.707 ± 0.094
	T2WI	0.817 ± 0.066	0.687 ± 0.114	0.961 ± 0.034	0.729 ± 0.093
	CE-FST1WI	0.807 ± 0.066	0.677 ± 0.148	0.958 ± 0.033	0.649 ± 0.096
	All	0.826 ± 0.065	0.689 ± 0.147	0.967 ± 0.027	0.707 ± 0.094
	Ensemble	0.808 ± 0.061	0.673 ± 0.139	0.960 ± 0.032	0.706 ± 0.098
LASSO (single) + SMOTE + RF	T1WI	0.820 ± 0.063	0.686 ± 0.127	0.949 ± 0.038	0.687 ± 0.093
	T2WI	0.820 ± 0.078	0.731 ± 0.126	0.957 ± 0.038	0.750 ± 0.095
	CE-FST1WI	0.800 ± 0.060	0.690 ± 0.127	0.954 ± 0.037	0.690 ± 0.086
	All	0.813 ± 0.066	0.715 ± 0.119	0.966 ± 0.030	0.697 ± 0.092
	Ensemble	0.813 ± 0.070	0.712 ± 0.126	0.958 ± 0.036	0.702 ± 0.096

**Table 4:** Performance of model with repeated feature selection

Model	Features	Accuracy (mean ± SD)	Kappa coefficient (mean ± SD)	AUC (mean ± SD)	mAP (mean ± SD)
LASSO (repeated) + RF	T1WI	0.807 ± 0.064	0.615 ± 0.174	0.947 ± 0.043	0.680 ± 0.093
	T2WI	0.820 ± 0.067	0.677 ± 0.134	0.956 ± 0.035	0.663 ± 0.100
	CE-FST1WI	0.801 ± 0.067	0.647 ± 0.147	0.950 ± 0.035	0.705 ± 0.090
	All	0.806 ± 0.056	0.657 ± 0.130	0.951 ± 0.034	0.687 ± 0.094
	Ensemble	0.805 ± 0.061	0.656 ± 0.146	0.961 ± 0.029	0.705 ± 0.097
LASSO (repeated) + SMOTE + RF	T1WI	0.801 ± 0.076	0.655 ± 0.176	0.946 ± 0.042	0.652 ± 0.093
	T2WI	0.815 ± 0.065	0.674 ± 0.152	0.949 ± 0.037	0.663 ± 0.100
	CE-FST1WI	0.802 ± 0.072	0.691 ± 0.105	0.946 ± 0.038	0.705 ± 0.090
	All	0.806 ± 0.062	0.681 ± 0.121	0.952 ± 0.036	0.687 ± 0.094
	Ensemble	0.806 ± 0.071	0.689 ± 0.126	0.951 ± 0.040	0.675 ± 0.099

Application of SMOTE generally did not lead to a substantial improvement in model performance. While slight increases in kappa coefficient and mean AP were observed in some combinations, overall accuracy and AUC values were not consistently enhanced.

In the second approach, where LASSO feature selection was applied at every iteration of the training set and validated in the test set. Compared with single application of LASSO, models using repeated LASSO generally demonstrated slightly decreased performance across all metrics. This trend was consistently observed regardless of the imaging feature sets or ensemble.

## Discussion

The main finding of our study is that the preoperative MRI based-radiomics model can effectively discriminate among different grades of cartilaginous neoplasm. Overall, performances across the various models were similar with highest accuracy and AUCs of LASSO (single) + RF from all sequences and LASSO, and highest mean AP of LASSO (single) + SMOTE + RF from T2WI.

The SMOTE algorithm was employed to address the class imbalance in our dataset by generating synthetic samples of the minority class [26]. Despite the theoretical advantage of balancing the training data, SMOTE did not consistently improve model performance in our study. A slight improvement in AP, which is considered a more appropriate performance metric for imbalanced datasets, was observed in some feature sets, particularly in T2WI-based models, suggesting that class imbalance may have had a greater impact in certain imaging method. However, the lack of consistent benefit across all models indicates that factors beyond simple class imbalance, such as feature quality and sample size, may play a more critical role in determining model performance. The repeated application of LASSO for feature selection during each fold of cross-validation was intended to minimize overfitting and enhance the generalizability of the models [28]. However, our results showed that repeated LASSO slightly degraded the model performance compared to a single application of LASSO. This may be due to the increased variability in feature selection across folds, leading to instability in model training.

Differentiating low-grade CSA from enchondroma remains a significant challenge, even for experienced radiologists and pathologists, due to the similarities in imaging and pathological features [4]. Accurate diagnosis is essential for determining the appropriate treatment options, which vary based on tumor grades [29]. While biopsy is a standard preoperative diagnostic tool, it is susceptible to sampling errors and may result in an underestimation of the diagnosis [11–13].

MRI has been widely used to evaluate cartilaginous neoplasm. However, previous studies have shown a variable range of inter-reader agreement for radiologic features [5, 30]. In light of this, there is a growing need for objective biomarkers that can enhance diagnostic accuracy. Radiomics, which involves the extraction of features from predefined formulas, texture analysis, and original or filtered images, holds promise for providing objective information independent of reader interpretation. However, the performance of radiomics models is heavily influenced by choices in preprocessing, feature selection, and classification [31, 32]. Previous studies have not fully explored variable methodological options to address data imbalance or improve model performance.

Human readers primarily rely on T2WI and contrast-enhanced sequence for interpretation of tumors, and do less on T1WI when interpreting cartilaginous tumors, focusing instead on its utility for detecting hemorrhage, fat content, or the intensity of edema. However, our study found that T1WI-based radiomics model, despite being underutilized by radiologists in this context, also provided fair performances. Previous studies also have similarly highlighted the importance of T1WI-derived texture features, particularly in differentiating enchondroma from low-grade CSA. Lisson et al. used texture analysis to discriminate low grade CSA from enchondroma and demonstrated that texture features from T1WI sequences were the most important features compared to those from T2WI. Texture features extracted from T2WI sequences showed no statistically difference between low grade CSA group and enchondroma group [33]. The authors reported that kurtosis in contrast-enhanced T1WI had the greatest power of differentiation, with 86% accuracy. Our result also showed that kurtosis was also selected from enhanced sequence. Fritz et al. graded cartilaginous bone tumors using 19 texture parameters [30]. The authors also reported that texture features extracted from T1WI sequence achieved high sensitivity and specificity for the differentiation of enchondroma and CSA. These findings suggest that T1WI holds substantial value in radiomics, offering insights that are not readily apparent to the naked eye.

Radiomics has been increasingly applied to the study of chondroid tumors, regardless of imaging modality. Previous studies have demonstrated the effectiveness of radiomics in differentiating between various grades of cartilaginous tumors, with models built on CT or MRI features showing varying degrees of success. Gitto et al. developed a machine learning classifier for differentiation atypical cartilaginous tumors and higher-grade CSA of long bones based on preoperative CT radiomic features. This study showed good accuracy in external test cohort and there was no statistical difference compared to accuracy of experienced radiologists [19]. Deng et al. reported that the CT texture analysis feature model could well differentiate cartilaginous tumors and could be the best model to differentiate between low- grade and higher-grade CSA. However, they reported that models with T1 and T2WI sequences derived features could not effectively differentiate low-grade from higher-grade CSA [12]. Our study contrasts with these works, particularly in the application of different methodological approaches like SMOTE and ensemble techniques. While past studies have shown mixed results depending on the modality and specific features used, our findings highlight the robustness of MRI-based radiomics, regardless of different sequence combinations and model structures.

Several limitations of our study need to be addressed. Firstly, this was a retrospective study, which may introduce biases inherent to such designs. Secondly, our study lacked an external validation cohort, which limits the generalizability of our findings. Thirdly, there was a notable class imbalance among the 3 groups, with significantly fewer CSA cases compared to enchondromas. Although we employed the SMOTE technique to address this issue, this cannot fully replicate the actual data distribution. Therefore, the potential impact of class imbalance on model generalizability cannot be completely addressed. Fourthly, we did not implement automatic segmentation techniques, which may limit the applicability of our model in routine clinical settings. Although manual segmentation allowed for precise delineation of tumor margins and exclusion of peritumoral edema, this approach is labor-intensive, time-consuming and prone to interobserver variability. Finally, diffusion-weighted imaging (DWI) was excluded from this study due to inconsistent availability and the presence of noise in some patients, despite previous reports suggesting limited utility of DWI in differentiating low-grade from high-grade CSA [34].

## Conclusions

In summary, we developed and assessed radiomics models for the 3-tier classification of cartilaginous bone tumors using preoperative MRI features and various methodological strategies. Across all model consistently showed fair to good performances, especially in models that utilized LASSO for feature selection and RF for classification.

## Data Availability

The primary datasets are not publicly available due to data privacy regulations. However, processed secondary data may be available from the corresponding author upon reasonable request.

## Abbreviations

AP: Average Precision
AUC: Area under the curveCSA Chondrosarcoma
DWI: Diffusion-weighted imaging
LASSO: Least absolute shrinkage and selection operator
RF: Random forest
ROI: Region of interest
SMOTE: Synthetic minority oversampling technique
TSE: Turbo spin echo
VOI: Volume of interest
WI: Weighted imaging
